# Multidimensional Assessment of Sarcopenia and Sarcopenic Obesity in Geriatric Patients: Creatinine/Cystatin C Ratio Performs Better than Sarcopenia Index

**DOI:** 10.3390/metabo14060306

**Published:** 2024-05-27

**Authors:** Mohamad Khalil, Agostino Di Ciaula, Nour Jaber, Roberta Grandolfo, Flavia Fiermonte, Piero Portincasa

**Affiliations:** Clinica Medica “A. Murri”, Department of Precision and Regenerative Medicine and Ionian Area (DiMePre-J), University of Bari “Aldo Moro”, 70124 Bari, Italy; mohamad.khalil@uniba.it (M.K.); agodiciaula@gmail.com (A.D.C.); n.jaber1@studenti.uniba.it (N.J.); r.grandolfo@studenti.uniba.it (R.G.); f.fiermonte1@studenti.uniba.it (F.F.)

**Keywords:** sarcopenia, bioelectrical impendence, creatinine/cystatin C ratio, sarcopenic index, sarcopenic obesity

## Abstract

The serum creatinine/cystatin C ratio (CCR) and the sarcopenia index (SI) are novel indicators for sarcopenia, but their accuracy may depend on various confounders. To assess CCR and SI diagnostic accuracy, we studied the clinical and biophysical parameters associated with sarcopenia or sarcopenic obesity. A total of 79 elderly patients (65–99 yrs, 33 females) underwent clinical, anthropometric, body composition, geriatric performance, and blood chemistry evaluation. The CCR and SI accuracy were assessed to identify sarcopenia. Sarcopenia was confirmed in 40.5%, and sarcopenic obesity in 8.9% of the subjects. Sarcopenic patients showed an increased Charlson comorbidity index, cardiovascular disease (CVD) rates and frailty, and decreased physical performance than non-sarcopenic subjects. Patients with sarcopenic obesity had increased body fat and inflammatory markers compared to obese subjects without sarcopenia. Sarcopenia was associated with a decreased CCR and SI. However, when the logistic regression models were adjusted for possible confounders (i.e., age, gender, Charlson comorbidity index, presence of CVD, and frailty score), a significant OR was confirmed for the CCR (OR 0.021, 95% CI 0.00055–0.83) but not for the SI. The AUC for the CCR for sarcopenia discrimination was 0.72. A higher performance was observed in patients without chronic kidney diseases (CKD, AUC 0.83). CCR, more than the SI, is a useful, non-invasive, and cost-effective tool to predict sarcopenia, irrespective of the potential confounders, particularly in subjects without CKD.

## 1. Introduction

Sarcopenia is a progressive skeletal muscle disorder characterized by the loss of muscle mass, strength, and function [1]. Although sarcopenia is linked with aging, this condition starts earlier in life [2]. Sarcopenia is influenced by external factors, such as physical inactivity, inadequate nutrition, chronic diseases, and hormonal changes, and generates adverse and long-term outcomes, such as falls, frailty, disability, and increased mortality. The diagnosis of sarcopenia relies on a different consensus, leading to varied prevalence rates characterized by heterogeneity [3]. Sarcopenic obesity is defined as a decreased lean body mass (muscle mass) associated with increased adiposity. Obesity worsens sarcopenia through an increase in fat infiltration into muscles, decreased physical activity, and increased mortality risk [4].

The European Working Group on Sarcopenia in Older People (EWGSOP) [5] identified low muscle strength as a key characteristic in the diagnosis of sarcopenia. The diagnosis is confirmed through an assessment of low muscle quantity and quality, while physical performance is considered as an indicator of the severity of sarcopenia. The assessment of sarcopenia involves the analysis of various indicators, including muscle strength (i.e., by dynamometer) [6,7], the amount of muscle assessed by magnetic resonance imaging (MRI), computed tomography (CT), dual-energy X-ray absorptiometry (DXA) or bioelectrical impedance analysis (BIA) [8,9,10,11], and physical performance (i.e., Short Physical Performance Battery (SPPB), Timed-Up and Go test (TUG), and the 400 m walk test, Gait Speed) [12].

A comprehensive panel of diagnostic tools is usually not easily accessible in primary health care due to the high cost of the examinations and the need for specialized personnel. Thus, the identification of adequate biomarkers or indices to predict the presence of sarcopenia has a relevant role in both the diagnosis and management of this condition [13,14]. Valid indices should explore the critical pathogenic pathways such as neuromuscular junction, muscle protein turnover, behavior and inflammation, oxidative stress, hormones, and specific anabolic elements [13,14].

The ratio of serum creatinine and serum cystatin C (creatinine/cystatin C, known as CCR) [15,16] as well as serum creatinine × eGFR_cystatin C_, known as the sarcopenia index (SI) [17], have been recently indicated as possible predictors of sarcopenia. In particular, a meta-analysis investigated the relationship between CCR and sarcopenia and the predictive value of CCR in hospitalized patients [15]. The results pointed to CCR as an effective screening tool for sarcopenia, with a diagnostic value (expressed as area under the curve, AUC) ranging between 0.6 and 0.81. Further evidence indicates that the SI is an index strongly associated with skeletal muscle mass and a simple and useful tool for assessing sarcopenia [18,19]. However, residual concerns exist since the diagnostic accuracy of these indices might depend on the presence of possible clinical confounders, in particular, in the presence of multiple comorbidities. Thus, the current use of these indices in fragile patients still requires comprehensive validation.

To verify the capability of the serum CCR and SI as valuable diagnostic tools for sarcopenia, we prospectively analyzed an Italian cohort of geriatric patients. We used a multidimensional clinical, physical, and biophysical approach to diagnose sarcopenia and sarcopenic obesity. Furthermore, since serum creatinine and cystatin C are commonly employed in clinical settings for evaluating renal function, the performance of the CCR and SI to predict sarcopenia was also investigated in patients with or without chronic kidney disease (CKD).

## 2. Materials and Methods

### 2.1. Patients

A total of 79 patients aged 65 years or older (46 males, 33 females) were recruited on a voluntary basis during outpatient visits at the Internal Medicine division “A. Murri”, AOUC Polyclinic, Bari, Italy, from March to September 2023. At enrolment, the subjects underwent a full clinical assessment, including a detailed medical history and physical examination, to identify possible exclusion criteria. Exclusion criteria were retirement for terminal conditions, a peripheral vascular disease with intermittent claudication, acute arthritis, relapsed chronic obstructive pulmonary disease with severe respiratory failure, acute heart failure (New York Heart Association class IV), peripheral edema/anasarca, acute malnutrition, presence of a pacemaker or an implanted cardiovascular defibrillator, and lack of informed consent. Demographic data, including age, sex, and smoking status, were collected as part of the initial patient examination. Additionally, comorbidities such as dyslipidemia, hypertension, diabetes mellitus, heart diseases, respiratory diseases, neoplasia, thyroid diseases, and chronic renal insufficiency were explored in all patients. These clinical details were recorded separately from subsequent instrumental procedures, which were performed by different, blinded professionals. All subjects signed a written informed consent before the beginning of the study. The study was approved by the local ethical board (Study number 7506, protocol number 0030591|28 March 2023).

### 2.2. Anthropometric Measurements

The anthropometric measurements included the following: height to the nearest 0.1 cm and weight to the nearest 0.1 kg; and waist, calf, and mid-upper arm circumferences to the nearest 0.1 cm. The body mass index (BMI) was calculated using the following formula: weight (kg)/height (m^2^). The circumferences were measured using a flexible, non-stretchable tape measure. The waist circumference (WC) was measured at the level of the iliac crest; the calf circumference (CC) was measured while the patient was seated with their knees at 90°, taking the calf’s greatest circumference; mid-upper arm circumference was taken (MAC) at the midpoint between the shoulder and the elbow of the dominant arm.

### 2.3. Hand Grip Test

Hand grip strength was assessed by a digital dynamometer (Kuptone brand, model EH101) on the dominant hand. The dynamometer provides an accurate momentary reading of digital grip power with a high-precision strain gauge sensor. The measurement capacity was as follows: 198 pounds/90 kg; division: 0.2 kg/100 g. The measurement was taken in triplicate, with a one-minute interval between each measurement. The average of the three measurements was thereafter calculated. We used the cut-offs established by EWGSOP for the definition of reduced muscle strength, i.e., 27 kg for males and 16 kg for females [5,20].

### 2.4. Assessment of Body Composition by Bioelectrical Impedance Analysis

The bioelectrical impedance analysis (BIA) was measured using a single-frequency device measured at 500 kHz (Body impedance analyzer, Nutribox, Pocking, Germany). During the examination, patients were positioned supine on an examination table with their arms extended alongside the body and their legs spread apart at an approximately 45-degree angle. Pairs of electrodes were attached to the wrist and extremity of the right hand, as well as to the ankle and extremity of the right foot. Throughout the examination, the patients were instructed to remain still to minimize movement artifacts. Preliminary patient information, including age, sex, height, weight, and abdominal circumference, was inserted into the BIA software (Nutribox, WinFood, Colonnella, Italy. The resistance(R), reactance (Xc), resistance index (RI, calculated as height (cm^2^)/R(Ω)), and phase angle (PhA) were provided by the software. Total body water (TBW, liter), body cell mass (BCM, Kg), body fat mass (BFM, kg, and %), basal metabolic rate (BMR, Kcal), extracellular mass (ECM, kg), lean mass (LM, kg), phase angle (PhA), and the percentage of active cells (%) were also provided.

### 2.5. Assessment of Geriatric Performance

The physical performance of the enrolled patients was assessed by the short physical performance battery (SPPB), which is a panel of tests used to assess balance, lower extremity strength, and functional capacity in older adults. The SPPB consists of balance assessment tests, the chair stand test, and the gait speed test (4-m) [21,22]. All tests were performed according to the guidelines mentioned in the references. The total score ranges from 0 to 12; a higher score indicates good lower limb function. Frailty was assessed according to Fried’s criteria, where a frailty phenotype is defined as the presence of three of the following criteria: unintentional weight loss, self-reported exhaustion, reduction in muscle strength (grip strength), low physical activity, and slow walking speed (gait speed test). Pre-frailty is present if only 2 criteria are present [23]. The patient’s degree of autonomy was studied using the Activities of Daily Living (ADL) and Instrumental Activities of Daily Living (IADLs) scales. The scale ranges from 0 to 8; a higher score depicts better functionality [24]. The Mini Nutritional Assessment (MNA) was used to assess the risk of malnutrition in elderly patients. It is a score-based questionnaire that depicts if patients have normal nutrition (score above 23.5) or suffer from malnutrition (score lower than 23.5) [25]. The Charlson comorbidity index (CCI) was estimated using an online calculator MDCalc Ltd. Inc. New York, NY 10003, USA (https://www.mdcalc.com/charlson-comorbidity-index-cci, accessed on 28 August 2023).

### 2.6. Blood Chemistry

Blood tests measured albumin (g/dL), pre-albumin (g/L), total protein (g/dL), C-reactive protein (CRP, mg/L), white blood cell counts (WBC, ×10^3^/μU), vitamin D (ng/mL), IL-6 (pg/mL), fasting glucose (mg/dL), insulin (μU/mL), glycated hemoglobin (HbA1c, mmol/mol), total cholesterol (mg/dL), low-density lipoprotein cholesterol (LDL, mg/dL), high-density lipoprotein cholesterol (HDL, mg/dL), triglycerides (TG, mg/dL), creatinine (mg/dL), estimated glomerular filtration rate estimated with creatinine (eGFR Cr, mL/min), cystatin (C, mg/mL), and eGFR estimated with cystatin C (eGFR Cis, mL/min). The homeostatic model assessment for insulin resistance (HOMA-IR) (blood glucose (mg/dL) × insulin (μU/mL)/405) was also calculated.

### 2.7. Diagnosis of Sarcopenia

According to EWGSOP2 [5], the diagnosis of probable sarcopenia was determined in the presence of low muscle strength. The diagnosis was confirmed with the additional presence of low muscle quantity or quality. Severe sarcopenia was diagnosed when low muscle strength, low muscle quantity/quality, and poor physical performance were all observed. Low muscle quantity was evaluated by using the appendicular skeletal muscle mass (ASSM) by applying Sergi’s equation: ASMM (kg) = −3.964 + (0.227 × RI) + (0.095 × weight) + (1.384 × sex) + (0.064 × Xc), where RI (resistance index) = height^2^ (cm)/R; Xc = reactance (ohms); sex: male = 1; female = 0. The cut-off values adopted for ASMM are those suggested by EWGSOP2, i.e., 20 kg for males and 15 kg for females. Sarcopenic obesity is detected when the percent of fat mass is >25% (men) and >35% (women) [26].

### 2.8. Calculation of Creatinine/Cystatin C Ratio (CCR) Score and Sarcopenic Index (SI)

The CCR index was calculated by dividing the serum creatinine levels by the serum cystatin C levels. A lower CCR score indicates lower muscularity and hence predicts a risk of sarcopenia. The sarcopenia index (SI) was calculated as follows: serum creatinine multiplied by the estimated eGFR with cystatin C (Cr × GFR_CysC_) [27].

### 2.9. Data Analysis

Data were presented as the mean ± standard error (SEM) for continuous normally distributed data or as a percentage for the categorical variables. Statistical significance between the two groups was determined by the Student’s t-test for the parametric data and the Mann–Whitney test for the nonparametric data. To compare more than 2 groups, the Kruskal–Wallis test followed by Dunn’s multiple comparison tests were used. Pearson’s chi-square was used to test the categorical variables; Fisher’s exact test was used if the sample size was small (<5). All statistical analyses were performed using NCSS 21 software (East Kaysville, USA). Logistic regression models were fitted using R software version 3.1.1 (The R Foundation for Statistical Computing, Vienna, Austria).

## 3. Results

### 3.1. General Characteristics of Subjects

Table 1 depicts the general characteristics of the enrolled patients. A total of 5.1% of the subjects were underweighted. In the majority of cases, a normal weight was recorded (38.0%). Subjects with overweight and obesity were 26.6% and 30.3%, respectively. Probable sarcopenia was diagnosed in 25.3% of subjects. Confirmed sarcopenia and sarcopenic obesity were present in 40.5% and 8.9% of subjects, respectively.

A total of 20.3% of the subjects were affected by malnutrition, and the majority of enrolled patients (77.2%) were fragile.

### 3.2. Assessment of Clinical, Physical, and Bio-Electric Parameters According to Sarcopenic Conditions

The demographic and clinical characteristics of the subjects, divided according to the diagnosis of sarcopenia, are detailed in Table 2. The sex rate and average BMI were comparable between subgroups. Subjects with confirmed sarcopenia were significantly older than non-sarcopenic subjects and showed an increased rate of cardiovascular diseases, Charlson comorbidity index, and frailty.

Table 3 shows the body composition of subjects (BIA) according to the diagnosis of sarcopenia. The BMR, phase angle, BCM, and percentage of metabolically active cells were significantly reduced in the sarcopenic group, as compared to probable sarcopenia and non-sarcopenic subjects. Values of TBW were significantly higher in sarcopenic patients than in subjects with probable sarcopenia and in the non-sarcopenic group. The lowest average lean mass was recorded in the non-sarcopenic subjects. The BFM and ECM were comparable between groups, and the ECM/BCM ratio was significantly higher in the sarcopenic, as compared with the non-sarcopenic group. Reactance was comparable between groups, but the resistance, resistance angle, and ASM were significantly lower in the sarcopenic, as compared with the probable sarcopenia group and non-sarcopenic group. These values were higher in subjects with probable sarcopenia, as compared with the non-sarcopenic group. The ASM/BMI ratio was significantly lower in subjects with sarcopenia as compared to the subjects with probable or absent sarcopenia.

**Figure 1:**
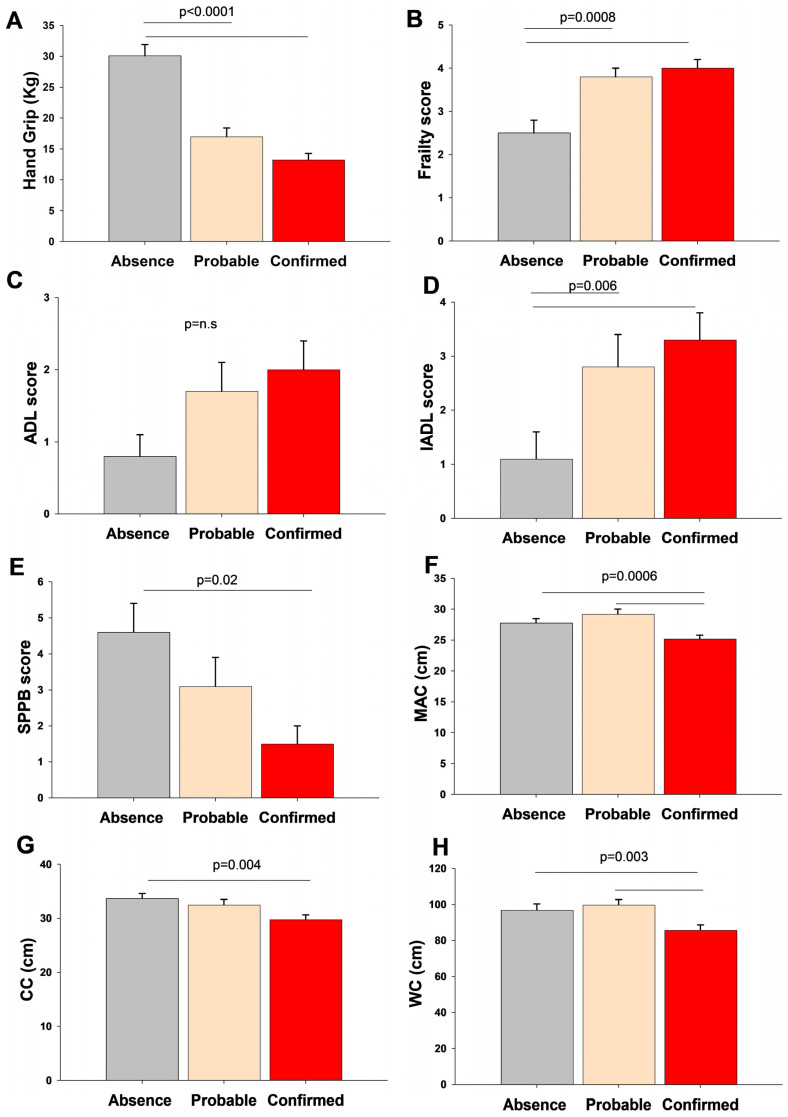
Differences in muscle strength (**A**), frailty score (**B**), physical performance scores (**C**,**D**), physical function (**E**), and anthropometric measurements (**F**–**H**) according to sarcopenia groups. Data are shown as bars (means) and error bars (standard error). The Kruskal–Wallis-Multiple comparison Z-value test (Dunn’s test) tested the difference between groups. Abbreviations: ADL, activities of daily living; CC, calf circumferences; IADL, Instrumental Activities of Daily Living; MAC, middle-upper arm circumference; SPPB, Short Performance Physical Battery; WC, waist circumferences.

We explored the differences in geriatric assessment according to the sarcopenia groups Figure 1. Sarcopenic subjects displayed reduced muscle strength, physical performance and function, and anthropometric measures with an increased frailty score than the non-sarcopenic patients.

All bio-humoral parameters were comparable among the subgroups except for albumin, pre-albumin, glycemia, cystatin C, and eGFR cys C (Table 4). In particular, albumin and pre-albumin were significantly lower in the sarcopenic than in the non-sarcopenic group. Fasting blood glucose was higher in the probable sarcopenia group than in the non-sarcopenic and sarcopenic groups. Cystatin C and the estimated glomerular filtration levels were significantly different in the non-sarcopenic group than in both the probable sarcopenic and sarcopenic subjects.

### 3.3. Role of CCR and SI in Predicting Sarcopenia

**Figure 2:**
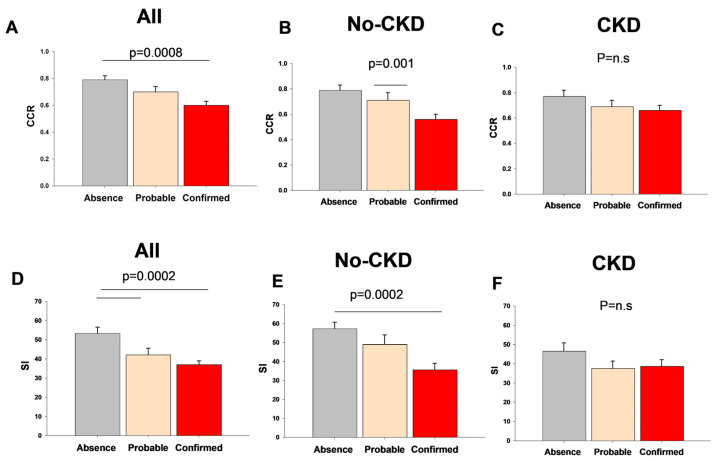
Variation in serum creatinine/serum cystatin C index (CCR) and sarcopenic index (SI) in the whole population and with or without chronic kidney disease (CKD) according to sarcopenia groups.

Figure 2 describes the variations in the CCR and SI indices according to the diagnosis of sarcopenia in the whole population and in patients with or without CKD.

The CCR and SI values in non-sarcopenic patients were 0.79 ± 0.03 and 53.4 ± 3.2, respectively, and significantly decreased in patients with confirmed sarcopenia (0.61 ± 0.03, *p* = 0.0008; 37.1 ± 1.9, *p* = 0.0002, respectively).

Among the subjects with sarcopenia, the SI and CCR values were comparable in the subjects with or without CVD (SI: 35.8 ± 2.1 and 43.9 ± 2.0; CCR 0.60 ± 0.03 and 0.67 ± 0.04, respectively, *p* = NS). In the same subgroup of subjects, both indices showed significantly higher values in the male gender, as compared with females (SI: males 41.9 ± 2.3, females 32.3 ± 2.5, *p* = 0.007; CCR: males 0.69 ± 0.04, females 0.53 ± 0.02, *p* = 0.0009).

When patients were divided according to the presence of CKD, comparable results were observed in the case of both CCR and SI in patients without CKD. This difference was not evident in patients with CKD.

**Figure 3:**
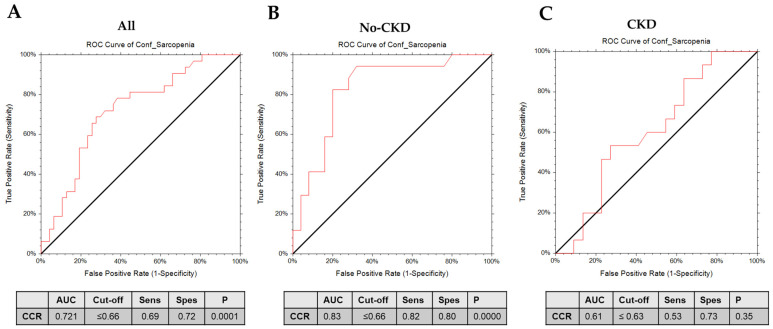
ROC curves of serum creatinine/serum cystatin C (CCR) in the diagnosis of sarcopenia in the (**A**) whole population, (**B**) non-CKD patients, and (**C**) CKD patients.

We fitted separate logistic regression models to calculate the odds ratios (OR) and confidence intervals (CI) for sarcopenia as independent variables associated with the measurement of the CCR or SI as dependent variables. In the crude model, the presence of sarcopenia was linked with a decreased CCR score (OR 0.01, 95%CI 0.0007–0.28) and with decreased SI score (OR 0.95, 95%CI 0.92–0.986). However, when the results were adjusted for the confounding effect of age, gender, Charlson comorbidity index, presence of cardiovascular diseases, and frailty score, a significant OR was only confirmed for the CCR (OR 0.021, 95%CI 0.00055–0.83) but not for the SI (OR 0.97, 95%CI 0.92–1.01). When the presence of sarcopenic obesity was confirmed, logistic regression models failed to confirm the significant associations between the presence of this condition and both the CCR (OR 0.09, 95%CI 0.001–8.86) and SI (OR 0.956, 95%CI 0.899–1.01). We, therefore, implemented a receiver operating characteristic (ROC) analysis to study the predictive capacity of CCR in distinguishing confirmed sarcopenia in the whole population and in patients with or without CKD (Figure 3). In the total population, the CCR showed fairly high predictive efficacy, indicated by the AUC values of 0.721 (*p* = 0.0001). Furthermore, a higher predictive power of the CCR was observed in non-CKD patients (AUC of 0.84 *p* < 0.00001). However, in patients with CKD, the AUC decreased for the CCR (AUC = 0.61, *p* = NS). Detailed information about the cut-offs, sensitivity, and specificity are reported in Figure 3.

### 3.4. Sarcopenic Obesity

Table 5 summarizes the findings in subjects with obesity or sarcopenic obesity. The two subgroups were comparable in age and BMI. When the BIA was assessed, the BMR, TBW, LM, BCM, ASM, and ASM/BMI ratio were significantly lower in subjects with sarcopenic obesity (*p* < 0.05). However, the BFM percentage was significantly higher in subjects with sarcopenic obesity than in those with obesity (*p* < 0.05). In the analysis of physical performance using the SPPB, a significantly higher score was recorded in patients with obesity without sarcopenia than in those with sarcopenic obesity. For physical function measures, both the ADL and IADL mean values were significantly higher in the sarcopenic obesity group than in the obese group. The serum levels of CRP and IL-6 were higher in the sarcopenic obesity group, while the serum glucose was significantly higher in patients with obesity without sarcopenia than in the sarcopenic obesity group. The CCR and SI indices were comparable between the two subgroups.

## 4. Discussion

This study evaluated the clinical, physical, and biophysical characteristics in a cohort of geriatric patients screened for sarcopenia and sarcopenic obesity. The results indicate that the CCR, more than the SI can be considered a useful, non-invasive, and cost-effective tool to predict sarcopenia, irrespective of the possible confounders, in particular in subjects without CKD.

The screening and diagnosis of sarcopenia are crucial for preventing adverse health consequences. The CT, MRI, and DXA serve as reference standards for non-invasively assessing muscle quantity/mass and detecting adipose tissue. However, these techniques are expensive and require specific hospital facilities. In addition, the use of BIA as an accurate diagnostic tool for detecting sarcopenia should be accompanied by a validated BIA equation and the use of functional and physical tests [28]. Our study focuses on evaluating the diagnostic efficacy of the CCR and SI since these indices can offer distinct advantages such as simplicity, non-invasiveness, and potential applicability in diverse clinical settings, as compared with other tools for the identification of sarcopenia. From a comparative perspective, the diagnosis of sarcopenia should include both well-established imaging modalities and emerging techniques, like BIA and the assessment of non-invasive biomarkers, to improve accessibility, cost-effectiveness, and comprehensive diagnostic accuracy.

In the cohort enrolled in the present study, the prevalence of sarcopenia was 40.5%. This rate was higher than the previously reported overall prevalence (between 10% and 27%, according to EWGSOP2 [29]). One explanation is that most patients (about 80%) were hospitalized and had different comorbidities (in particular CVD, 85%). Recent studies underscored a longitudinal link between the high prevalence of CVD and sarcopenia or probable sarcopenia among middle-aged and elderly individuals [30,31,32]. Our findings are in line with this evidence. In the present cohort, however, the high prevalence of CVD among subjects with sarcopenia could be considered a relevant confounder in determining the role of the SI and CCR. However, no difference emerged in both the SI and CCR in sarcopenic subjects with or without CVD. Furthermore, when the links between these indices and sarcopenia were examined by logistic regression analyses, the models were adjusted for the confounding effect of CVD.

In the comparison of sarcopenic and non-sarcopenic patients, notable differences emerged across the various parameters. Sarcopenic patients exhibit a significantly lower phase angle and percentage of metabolically active cells compared to non-sarcopenic patients. Phase angle, reflecting cell membrane functionality, tends to be lower in sarcopenic patients, indicating potential health challenges [33,34]. Anthropometric measurements also reveal distinctions, with sarcopenic individuals displaying lower mid-arm circumference (MAC) and calf circumference (CC), which are indicative of reduced muscle mass [35]. Sarcopenic patients tend to have lower levels of albumin and pre-albumin, potentially affecting nutritional status and muscle mass [36].

In this study, 8.9% of the patients had sarcopenic obesity, in line with the overall prevalence of sarcopenic obesity in the elderly (about 11%) [37]. Of note, the results from the logistic regression models indicate that the use of the CCR and SI should not be accurate in subjects with sarcopenic obesity. Thus, further and specific studies are needed to possibly identify useful biomarkers to be applied in patients with sarcopenic obesity. Our results also point to the critical role of diagnosing this specific condition, which can act as a relevant confounder in the use of biomarkers for sarcopenia.

At the same BMI, subjects with sarcopenic obesity had different body composition parameters than individuals with obesity and without sarcopenia. These results reinforce the concept that some current diagnostic criteria (based on an obesity diagnosis only through BMI) are inaccurate, as concomitant loss of muscle mass and an increase in fat may result in a negligible or null net change in body weight or BMI. This also means that many sarcopenic subjects (apparently non-obese) could actually be sarcopenic obese subjects who have not been suspected and/or tested for (pre-)sarcopenia, and we might significantly underestimate both the prevalence and impact of sarcopenic obesity. Indeed, visceral obesity is correlated with appendicular muscle loss [4]. In this context, chronic inflammation in obesity may lead to systemic atrophy and the wasting observed in cachexia and full sarcopenia. Consistent with this, we observed significantly higher values of inflammation markers, particularly tripled values of CRP and almost doubled values of IL-6, in sarcopenic obese subjects compared to the group of obese patients without sarcopenia. This observation is in line with current evidence suggesting the high expression of inflammatory markers in sarcopenic obesity patients with a negative influence on muscle strength and sarcopenia [38,39,40,41,42,43].

Recent evidence indicates that biomarkers and indices for sarcopenia offer a proper diagnosis tool for sarcopenia. Our data underline that it is crucial to investigate the potential role of those biomarkers using a comprehensive and multidimensional approach to accurately check the role of possible confounders.

The CCR and SI, in particular, have been widely validated as readily available and reproducible biomarkers across diverse populations [44]. Several studies established their correlation with skeletal muscle mass, measured through computed tomography (CT) or dual-energy X-ray absorptiometry (DEXA) [17,45].

The CCR shows promise as a valuable predictor for various adverse outcomes, including treatment-related side effects, fractures, hospitalizations, and mortality among intensive care unit patients [46].

In the present study, the CCR, but not the SI, was associated with the presence of sarcopenia independently from possible confounders. The CCR showed high predictive efficacy for sarcopenia discrimination (AUC = 0.72), also including subjects with CKD.

A recent meta-analysis [47] revealed that the CCR had an AUC diagnostic value for low muscle mass according to the EWGSOP2 diagnostic criteria of 0.70 and 0.63 for males and females, respectively. Similarly, another meta-analysis [15] reported the AUC value for the CCR in predicting sarcopenia diagnosed by CT, which ranged between 0.6 and 0.8.

All studies regarding the predictive capability of the CCR and SI for sarcopenia were conducted on Asian ethnic populations, often with the selected sample entirely affected by neoplastic diseases, diabetes mellitus, or chronic obstructive pulmonary diseases. In our study, the enrolled cohort is heterogeneous for the presence of a number of comorbidities. In addition, all studies reported the use of CT or DEXA as the gold standard reference, while in our study, we used BIA. In addition, we separately explored the diagnostic accuracy of the CCR by ROC analysis in patients with or without CKD. We observed a higher AUC value of 0.82 in individuals without CKD, indicating better discriminative ability in this subgroup. This suggests that CKD may indeed serve as a relevant confounder in the evaluation of the CCR as a diagnostic marker for sarcopenia. To our knowledge, the only previous study focusing on the association between the cystatin C to creatinine ratio C/Cr (the inversion of the CCR) and sarcopenia in non-dialysis-dependent CKD [48] showed a moderately valuable diagnostic performance of the C/Cr ratio for sarcopenia (AUC = 0.656). Our findings are in line with previous evidence that highlights the intricate relationship between CKD and muscle wasting [49], which could potentially affect the utility of the CCR as a diagnostic tool. CKD, in particular, has been associated with altered muscle metabolism, including increased protein catabolism and reduced muscle mass [50]. Furthermore, factors such as inflammation, oxidative stress, and hormonal imbalances are commonly observed in patients with CKD and may contribute to the observed differences in the diagnostic accuracy of the CCR. Thus, while our study highlights the potential utility of the CCR in predicting sarcopenia, the critical role of CKD as a relevant confounder should be carefully taken into account.

This study has some limitations, such as the limited sample size of the examined population and the scarce number of subjects with sarcopenic obesity. This last limitation is of particular relevance since sarcopenic obesity represents a distinct subgroup of patients within the spectrum of sarcopenia. Thus, further studies should include a larger cohort of individuals with different phenotypes, including an adequate number of subjects with sarcopenic obesity. Furthermore, although the results from logistic regression models point to the CCR as a significant predictor of sarcopenia independent from gender, further studies specifically aimed to explore the possible gender differences in the routine use of the CCR are certainly needed. Data from the present study, however, point to a clear role for the CCR in possibly predicting the presence of sarcopenia also in subjects with possible confounding effects, which are played by frailty and multiple comorbidities (in particular, CVD and CKD).

On the other hand, the precise, possible roles of the CCR and SI in real life and the current clinical practice should be better assessed in future studies, with a comprehensive consideration for multiple individual confounders rather than in selected populations.

## 5. Conclusions

The assessment of indices being possibly predictive for sarcopenia should be based on a multidimensional analysis involving a heterogeneous group of subjects and should consider a comprehensive panel of clinical, anthropometric, biochemical, and biophysical parameters. In fact, sarcopenia represents a multifaceted syndrome that is associated with various systemic diseases and requires a multi-level approach for accurate diagnosis and effective management.

In this scenario, the results from the present study point to the CCR, but not to the SI, as a reliable tool to predict sarcopenia diagnosis in clinical practice.

A relevant additional finding is the existence of a potential disparity in the predictive efficacy among patients with or without CKD, highlighting the need for further investigation in this domain.

Finally, these data point to the need for an accurate and comprehensive diagnosis and characterization of subjects with sarcopenic obesity to allow for the best management of disease in the presence of this specific and frequently underdiagnosed clinical condition.

## Figures and Tables

**Table 1:** Demographic and clinical characteristics of 79 patients enrolled in this study.

General Characteristics	Total (N = 79)	Male (N = 33)	Female (N = 46)	*p*
Age (years)	78.2 ± 0.9	79.2 ± 1.6	77.4 ± 1.0	0.3
BMI (kg/m^2^)	26.9 ± 0.7	29.1 ± 1.2	25.4 ± 0.9	0.009
Underweight N (%)	4 (5.1%)	1 (3.0%)	3 (6.5%)	0.48
Normal weight N (%)	30 (38.0%)	7 (21.2%)	23 (50.0%)	0.009
Overweight N (%)	21 (26.6%)	8 (24.2%)	13 (28.3%)	0.69
Obese N (%)	24 (30.3%)	17 (51.5%)	7 (15.2%)	0.0005
Smoker N (%)	5 (6.3%)	2 (6.1%)	3 (6.5%)	0.93
Former smokers N (%)	30 (38.0%)	6 (18.2%)	24 (52.2%)	0.002
Comorbidities N (%)				
Dyslipidemia	50 (63.3%)	23 (69.7%)	27 (58.7%)	0.32
Arterial Hypertension	57 (72.15%)	24 (72.7%)	33 (71.7%)	0.92
Dysthyroidism	21 (26.6%)	13 (39.4%)	8 (17.4%)	0.03
Atheroma	29 (36.7%)	9 (27.3%)	20 (43.5%)	0.1
Diabetes	27 (34.2%)	10 (30.3%)	17 (37.0%)	0.53
Cardiovascular Disease	55 (69.6%)	21 (63.6%)	34 (73.9%)	0.32
Respiratory Disorders	47 (59.5%)	17 (51.5%)	30 (65.2%)	0.22
Neoplasms	31 (39.2%)	8 (24.2%)	17 (40.0%)	0.27
Chronic renal disease	37 (64.6%)	17 (51.5%)	20 (43.5%)	0.48
Charlson Comorbidity Index	8.2 ± 0.2	8.2 ± 0.4	8.2 ± 0.3	0.97
Sarcopenia N (%)				
No sarcopenia	27 (34.2%)	8 (24.2%)	19 (41.3%)	0.29
Probable Sarcopenia	20 (25.3%)	9 (27.3%)	11 (23.9%)	0.29
Confirmed Sarcopenia	32 (40.5%)	16 (48.5%)	16 (34.8%)	0.29
Sarcopenic obesity	7 (8.9%)	5 (15.2%)	2 (4.3%)	0.1
Malnutrition N (%)				
No malnutrition	31 (39.2%)	12 (36.4%)	19 (41.3%)	0.9
Risk of malnutrition	32 (40.5%)	14 (42.4%)	18 (39.1%)	0.9
Malnutrition	16 (20.3%)	7 (21.2%)	9 (19.6%)	0.9
Fragility N (%)				
Non-fragile	3 (3.8%)	1 (3.0%)	2 (4.3%)	0.14
Pre-fragile	15 (19.0%)	5 (15.2%)	10 (21.7%)	0.14
Fragile	61 (77.2%)	27 (81.8%)	34 (73.9%)	0.14

All categorical data are presented as numbers (percent). Age, BMI, and Charlson comorbidity index are expressed as mean ± standard error. The T-test shows the difference between groups (age, BMI, Charlson comorbidity index). Pearson’s chi-squared test was used for categorical data. Abbreviations: BMI, body mass index; N, number of subjects.

**Table 2:** Demographic and clinical characteristics of 79 patients according to sarcopenia groups.

	Absence of Sarcopenia (N = 27)	Probable Sarcopenia (N = 20)	Confirmed Sarcopenia (N = 32)	*p*-Value
Females N (%)	8 (29.6%)	9 (45%)	16 (50%)	0.2
Age (years)	74.7 ± 1.5 *	78.3 ± 1.5	81.0 ± 1.4 *	0.01
BMI (kg/m^2^)	26.3 ± 1.1	29.7 ± 1.7	25.8 ± 1.0	0.13
Underweight N (%)	2 (7.4%)	0 (0.0%)	2 (6.3%)	0.1
Normal weight N (%)	8 (29.6%)	8 (40.0%)	14 (43.8%)	0.1
Overweight N (%)	11 (40.7%)	10 (10.0%)	8 (25.0%)	0.1
Obese N (%)	6 (22.2%)	20 (50.0%)	8 (25.0%)	0.1
Comorbidities N (%)				
Dyslipidemia	18 (66.7%)	11 (55.0%)	21 (65.6%)	0.6
Arterial Hypertension	18 (66.7%)	14 (70.0%)	25 (78.1%)	0.6
Thyroid disease	4 (14.8%)	5 (25.0%)	12 (37.5%)	0.1
Atheroma	13 (48.2%)	7 (35.0%)	9 (28.1%)	0.2
Diabetes	8 (29.6%)	10 (50.0%)	9 (28.1%)	0.2
Cardiovascular diseases	14 (51.9%)	14 (70.0%)	27 (84.4%)	0.02
Respiratory diseases	17 (63%)	9 (45.0%)	21 (65.6%)	0.3
Chronic kidney disease	13 (48.2%)	15 (75.0%)	32 (71.9%)	0.08
Neoplastic disease	11 (40.7%)	6 (30.0%)	14 (43.8%)	0.4
Charlson Comorbidity Index	7.3 ± 0.3 *	8.3 ± 0.7	8.9 ± 0.3 *	0.005
Malnutrition N (%)				
No malnutrition	15 (19.0%)	5 (6.3%)	11 (13.9%)	0.2
Risk of malnutrition	9 (11.4%)	10 (12.7%)	13 (16.5%)	0.2
Malnutrition	3 (3.8%)	5 (6.3%)	8 (10.1%)	0.2
Frailty N (%)				
Non-fragile	3 (3.8%)	0 (0.0%)	0 (0.0%)	0.01
Pre-fragile	10 (12.7%)	2 (2.5%)	3 (3.8%)	0.01
Fragile	14 (17.7%)	18 (22.8%)	29 (36.7%)	0.01

All categorical data are presented as numbers (percent). Age, BMI, and Charlson comorbidity index are expressed as mean ± standard error. The Kruskal–Wallis-Multiple comparison Z-value test (Dunn’s test) tested the difference between groups (age, BMI, Charlson comorbidity index), and similar symbol (Asterisk *) indicates statistical significance between groups. Pearson’s chi-squared test was used for categorical data. Abbreviations: BMI, body mass index; N, number of subjects.

**Table 3:** Bioelectric impedance parameters in the total population and according to sarcopenia groups.

	Total Population (N = 79)	Absence of Sarcopenia (N = 27)	Probable Sarcopenia (N = 20)	Confirmed Sarcopenia (N = 32)	*p*-Value
BMR (Kcal)	1282.9 ± 31.8	1358.9 ± 49.6 *	1420.0 ± 79.6 #	1133.1 ± 29.2 #,*	0.00003
PhA	4.6 ± 0.3	5.1 ± 0.4 *	5.4 ± 0.8 #	3.7 ± 0.2 #,*	0.003
TBW (L)	42.1 ± 4.3	39.0 ± 1.6 *	43.2 ± 1.2 #	44.1 ± 10.7 #,*	0.00008
LM (kg)	57.0 ± 5.9	53.3 ± 2.1 *,#	59.0 ± 1.7 #	58.8 ± 14.6 *	0.00001
ECM (kg)	30.0 ± 1.1	29.8 ± 1.9	33.6 ± 2.9	27.9 ± 1.2	0.2
BCM (kg)	21.4 ± 1.0	23.9 ± 1.3 *	25.6 ± 2.5 #	16.6 ± 0.9 #,*	0.00004
ECM/BCM	2.7 ± 1.0	1.6 ± 0.4 *	5.3 ± 3.9	1.9 ± 0.2 *	0.008
Percentage of Cells	41.3 ± 1.5	45.2 ± 2.2 *	43.7 ± 4.0 #	36.6 ± 1.5 #,*	0.003
BFM (%)	27.7 ± 1.4	26.5 ± 2.2	24.8 ± 3.4	30.3 ± 2.1	0.29
BFM (kg)	25.2 ± 2.3	20.5 ± 2.2	20.5 ± 2.6	21.0 ± 2.1	0.99
R (Ω)	584.2 ± 14.9	538.9 ± 21.0 $,*	437.5 ± 25.0 $,#	625.1 ± 19.6 #,*	0.00001
Xc (Ω)	42.8 ± 2.2	47.4 ± 4.4	42.8 ± 5.2	39.1 ± 2.2	0.36
RI (cm^2^/Ω)	52.5 ± 1.7	54.1 ± 2.5 $,*	65.2 ± 3.3 $,#	43.3 ± 1.7 #,*	0.00000
ASM (kg)	18.4 ± 0.5	19.3 ± 0.8 $,*	21.8 ± 0.9 $,#	15.4 ± 0.5 #,*	0.00000
ASM/BMI	0.7 ± 0.0	0.7 ± 0.0 *	0.8 ± 0.0 #	0.6 ± 0.0 #,*	0.004

Data were expressed as mean ± standard error. The difference between groups was tested by the Kruskal–Wallis-Multiple comparison Z-value test (Dunn’s test). Similar symbols (*,#,$) indicate statistical significance between groups. Abbreviations: ASM, appendicular skeletal mass; BCM, body cell mass; BFM, body fat mass; BMI, body mass index; BMR, basal metabolic rate; ECM, extracellular mass; LM, lean mas; N, number of subjects; PhA, phase angle; R, resistance; RI, resistive index (height^2^/resistance); Xc, reactance.

**Table 4:** Bio-humoral indices in the enrolled subjects divided according to diagnosis of sarcopenia.

	Total Population (N = 79)	Absence of Sarcopenia (N = 27)	Probable Sarcopenia (N = 20)	Confirmed Sarcopenia (N = 32)	*p*-Value
Albumin (g/dL)	5.7 ± 0.8	6.0 ± 1.4 $,*	7.4 ± 2.2 $	4.3 ± 1.0 *	0.007
Pre-albumin (g/L)	0.18 ± 0.02	0.22 ± 0.02 *	0.17 ± 0.02	0.15 ± 0.02 *	0.049
Total protein (g/dL)	12.3 ± 1.9	10.5 ± 2.8	12.3 ± 3.6	13.9 ± 3.4	0.06
WBC (×10^3^/μU)	96.7 ± 31.7	75.3 ± 40.9	215.6 ± 100.7	40.6 ± 28.1	0.90
CRP (mg/L)	62.4 ± 19.6	112.7 ± 56.6	29.3 ± 9.5	42.2 ± 7.9	0.12
IL-6 (pg/mL)	51.4 ± 11.0	60.9 ± 28.2	45.4 ± 16.1	47.0 ± 8.9	0.30
Glycemia (mg/dL)	97.3 ± 2.6	93.3 ± 3.4 $	110.1 ±6.0 $,#	92.8 ± 3.9 #	0.01
Insulinoma (μU/mL)	8.5 ± 0.6	10.3 ± 1.3	7.7 ± 1.0	7.5 ± 0.8	0.15
HOMA-IR	2.1 ± 0.2	2.4 ± 0.3	2.2 ± 0.3	1.8 ± 0.2	0.15
HbA1c (mmol/mol)	40.4 ± 1.6	37.5 ± 1.2	41.9 ± 4.5	41.9 ± 2.7	0.80
Creatinine (mg/dL)	1.1 ± 0.1	1.0 ± 0.1	1.3 ± 0.1	1.1 ± 0.1	0.27
eGFR_Cr_ (mL/min)	65.5 ± 2.8	73.0 ± 4.5	57.4 ± 5.9	64.3 ± 4.2	0.08
Cystatin C (mg/mL)	1.7 ± 0.1	1.4 ± 0.1 $,*	1.9 ± 0.2 $	1.7 ± 0.1 *	0.004
eGFR_Cys_ (mL/min)	47.6 ± 2.8	61.8 ± 5.2 $,*	40.3 ± 5.2 $	40.2 ± 3.1 *	0.004
25 (OH) Vit D (ng/mL)	18.4 ± 1.6	21.8 ± 2.8	17.4 ± 2.9	16.1 ± 2.4	0.12
Cholesterol (mg/dL)	135.4 ± 4.8	137.2 ± 9.6	128.9 ± 7.7	137.9 ± 7.3	0.66
LDL (mg/dL)	81.9 ± 4.0	82.5 ± 7.9	76.8 ± 6.7	84.7 ± 6.1	0.73
HDL (mg/dL)	35.2 ± 1.6	37.3 ± 2.7	32.9 ± 3.4	34.8 ± 2.6	0.40
Triglyceride (mg/dL)	11.1 ± 5.3	113.9 ± 11.1	112.1 ± 10.8	108.1 ± 6.2	0.99

Data were expressed as mean ± standard error. The Kruskal–Wallis-Multiple comparison Z-value test (Dunn’s test) tested the difference between groups. Similar symbols (*,#,$) indicate statistical significance between groups. Abbreviations: CRP, C-reactive protein; eGFR_Cr_, estimated glomerular filtration rate estimated with creatinine; eGFR_Cys_, estimated glomerular filtration rate estimated with cystatin; 25 (OH) Vit D, vitamin D; HbA1C, glycated hemoglobin; HDL, high-density lipoprotein cholesterol; HOMA-IR, homeostatic model assessment for insulin resistance; LDL, low-density lipoprotein cholesterol; N, number of subjects; WBC, white blood cells.

**Table 5:** Clinical characteristics, bioelectrical impedance, physical performance, physical function, and bio-humoral measures according to obesity and sarcopenic obesity.

	Obese (N = 17)	Sarcopenic Obesity (N = 7)	*p*
Age (years)	77.6 ± 1.7	85.0 ± 3.0	0.05
BMI (kg/m^2^)	34.8 ± 1.5	33.2 ± 0.8	0.5
BIA measurements			
BMR (Kcal)	1396.5 ± 53.0	1144.3 ± 41.1	0.008
PhA	4.8 ± 0.4	3.6 ± 0.2	0.1
TBW (L)	42.9 ± 1.9	32.9 ± 1.3	0.001
LM (kg)	55.8 ± 3.0	44.9 ± 1.8	0.007
BCM (kg)	24.7 ± 1.7	16.7 ± 1.3	0.01
BFM (%)	33.8 ± 2.4	45.0 ± 1.9	0.004
BFM (kg)	29.6 ± 2.5	36.8 ± 1.9	0.05
ASM	21.0 ± 1.1	15.4 ± 0.8	0.004
ASM/BMI	0.6 ± 0.02	0.5 ± 0.02	0.003
Physical measures			
SPPB	3.7 ± 0.9	0.0 ± 0.0	0.01
ADL	1.9 ± 0.5	4.0 ± 0.7	0.08
IADL	2.8 ± 0.7	5.7 ± 0.7	0.02
Bio-Humoral measures			
CRP (mg/L)	17.11 ± 3.8	62.3 ± 22.1	0.005
IL-6 (pg/mL)	26.0 ± 7.1	41.7 ± 16.0	0.04
Glycemia (mg/dL)	110 ± 6.4	87.9 ± 4.0	0.02
Creatinine (mg/dL)	1.07 ± 0.1	1.02 ± 0.1	0.6
Cystatin C (mg/mL)	1.7 ± 0.3	1.6 ± 0.1	0.5
Calculated indexes			
CCR index	0.68 ± 0.1	0.62 ± 0.1	0.5
SI index	42.9 ± 4.0	35.8 ± 3.2	0.3

Data were expressed as mean ± standard error. The difference between groups was tested by the Kruskal–Wallis-Multiple comparison Z-value test (Dunn’s test). Abbreviations: ADL: activities of daily living; ASM, appendicular skeletal mass; BCM, body cell mass; BFM, body fat mass; BMI, body mass index; BMR, basal metabolic rate; CCR, creatinine/cystatin C ratio; CRP, C-reactive protein; IADL, Instrumental Activities of Daily Living; LM, lean mas; N, number of subjects; PhA, phase angle; SI, sarcopenia index; SPPB, Short Performance Physical Battery.

## Data Availability

The data presented in this study are available upon request from the corresponding author.
